# The Effect of Urban Heat Island on Climate Warming in the Yangtze River Delta Urban Agglomeration in China

**DOI:** 10.3390/ijerph120808773

**Published:** 2015-07-27

**Authors:** Qunfang Huang, Yuqi Lu

**Affiliations:** 1 College of Geography Sciences, Nanjing Normal University, Wenyuan Road, Xianlin University District, Nanjing, 210023, China; 2 Jiangsu Center for Collaborative Innovation in Geographical Information Resource Development and Application, Nanjing 210046, China

**Keywords:** urban heat island, warming rate, urban agglomeration, Yangtze River Delta, urbanization rate, population, built-up areas

## Abstract

The Yangtze River Delta (YRD) has experienced rapid urbanization and dramatic economic development since 1978 and the Yangtze River Delta urban agglomeration (YRDUA) has been one of the three largest urban agglomerations in China. We present evidence of a significant urban heat island (UHI) effect on climate warming based on an analysis of the impacts of the urbanization rate, urban population, and land use changes on the warming rate of the daily average, minimal (nighttime) and maximal (daytime) air temperature in the YRDUA using 41 meteorological stations observation data. The effect of the UHI on climate warming shows a large spatial variability. The average warming rates of average air temperature of huge cities, megalopolises, large cities, medium-sized cities, and small cities are 0.483, 0.314 ± 0.030, 0.282 ± 0.042, 0.225 ± 0.044 and 0.179 ± 0.046 °C/decade during the period of 1957–2013, respectively. The average warming rates of huge cities and megalopolises are significantly higher than those of medium-sized cities and small cities, indicating that the UHI has a significant effect on climate warming (*t*-test, *p* < 0.05). Significantly positive correlations are found between the urbanization rate, population, built-up area and warming rate of average air temperature (*p* < 0.001). The average warming rate of average air temperature attributable to urbanization is 0.124 ± 0.074 °C/decade in the YRDUA. Urbanization has a measurable effect on the observed climate warming in the YRD aggravating the global climate warming.

## 1. Introduction

Since the beginning of the industrial revolution, industrial and agricultural activities, such as fossil fuel burning and land use change, have significantly increased the concentrations of greenhouse gases (GHG), such as carbon dioxide (CO_2_), methane (CH_4_), ozone (O_3_), nitrous oxide (N_2_O), and chlorofluorocarbons [1]. For example, since the beginning of the industrial era, the release of CO_2_ from human activities has resulted in atmospheric CO_2_ concentrations approximately increasing from 280 ppm to about 392 ppm in 2012 [1]. The 2013 IPCC report shows that global surface warming based on land and marine data was approximately 0.85 °C from 1880 to 2012 due to greenhouse effect. Additionally, the period of 1983–2012 was very likely the warmest 30-year period of the last 800 years and likely the warmest 30-year period of the last 1400 years [1]. Such an increase in air temperature results in rising sea levels, extreme weather events, complex and profound changes in terrestrial and aquatic ecosystems, altered modes of production, and altered lifestyles, thus forcing humans to adapt [2,3,4].

Because climate warming will affect human health in many ways through affecting surrounding environment and natural and social ecosystems, it is vital to describe the formation mechanism of climatic variations on regional to local scales. Cities have a warmer climate than rural areas due to the urban heat island (UHI), which represents one of the most significant human-induced changes to earth’s surface climate. The UHI effect has received considerable attention in climate warming research and in urban settlements [5,6,7,8,9]. Oke [10] ever reported that, even in a 1000 people town, an UHI effect could be observed, and the intensity of UHI is linearly correlated with the logarithms of the population, which is confirmed by other studies [11]. Because cities contain more than half of the world’s population [2], and because an estimated 70% of the global population is projected to live in cities by 2050 [12], city warming and heat waves due to the UHI effect have a profound impact on the lives, well-being and human health of urban residents [4,13,14].

On the global scale, urban area only occupies less than 0.5% of the Earth’s total land area based on the remote sensing estimation using 500 m spatial resolution Moderate Resolution Imaging Spectroradiometer (MODIS) [15]. Therefore, the influence of UHI on climatic change is thought to be very small. The IPCC [1] noted that it was unlikely that any uncorrected UHI effects and land use change effects would raise the estimated centennial globally averaged land surface air temperature trends by more than 10%. However, the IPCC [1] also admitted that 10% was an average value; in some regions with rapid development, the UHI and land use change impacts on regional trends might be substantially larger [16]. Schneider *et al.* [15] also pointed out that although only a small percentage of global land cover, urban areas could significantly alter climate at local, regional, and even global scales.

In China, there are also many studies on the warming effects of the UHI on the entire country or specific regions [5,17,18]. However, the results are inconsistent or even contradictory. Le *et al*. [17] reported that the average UHI effect for China from 1951 to 2001 was less than 0.01 °C/decade, suggesting that we cannot conclude that urbanization during the last 50 years has had an obvious effect on the observed warming in China. In contrast, Zhou *et al*. [5] suggested that climate warming of 0.05 °C/decade is attributable to urbanization in China, indicating a significant urbanization effect on climate warming. The warming rate due to urbanization reached 0.398 °C/decade, as detected by subtracting reanalysis data from observations, and 0.285 °C/decade, as determined by subtracting rural area values from urban area values, in East China from 1981 to 2007 [19]. The difference of warming rate in different studies in China was attributed to the different periods, different area and stations, and different variables (mean, maximum, and minimum air temperature). In addition, most studies only reported the warming trend and the UHI contribution to the warming rate. The driving factors and the potential mechanism of the UHI, for example, land use change from non-construction land to construction land, urbanization rate and population, in relation to climate warming are seldom interpreted [20,21]. Since the implementation of the policy of reform and opening up in late 1978, the Yangtze River Delta has experienced rapid urbanization and the Yangtze River Delta urban agglomeration (YRDUA) has been one of the three largest national urban agglomerations in China [22]. The effect of the UHI might be substantially larger in this region; thus, it cannot be neglected [23,24]. Therefore, more studies are needed to assess the contribution of the UHI effect to the warming rate and to discuss the causes of the UHI.

The aims of this study are to (1) present the climate warming rate for the YRDUA; (2) compare the warming rates for different urban sizes; and (3) qualitatively assess the factors that affect the warming rate and differentiate the UHI contributions to the warming rate using 41 meteorological stations observation data in the Yangtze River Delta (YRD) region.

## 2. Experimental Section

### 2.1. Study Area

**Figure 1:**
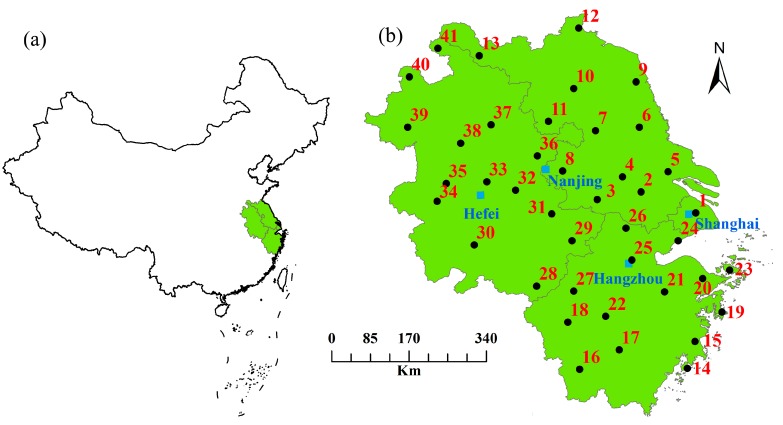
Locations of the YRDUA (**a**) and the 41 meteorological stations (**b**). 1: Longhua; 2: Wuxi; 3: Liyang; 4: Changzhou; 5: Nantong; 6: Dongtai; 7: Gaoyou; 8: Nanjing; 9: Sheyang; 10: Huai’an; 11: Xuyi; 12: Ganyu; 13: Xuzhou; 14: Yuhuan; 15: Hongjia; 16: Longquan; 17: Lishui; 18: Quzhou; 19: Shipu; 20: Yinxian; 21: Shengxian; 22: Jinhua; 23: Dinghai; 24: Pinghu; 25: Hangzhou; 26: Huzhou; 27: Chun’an; 28: Tunxi; 29: Ningguo; 30: Anqing; 31: Wuhu; 32: Chaohu; 33: Hefei; 34: Huoshan; 35: Lu’an; 36: Chuxian; 37: Bengbu; 38: Shouxian; 39: Fuyang; 40: Bozhou; and 41: Dangshan.

YRD is located along the central-eastern coastline of China, comprising Shanghai, southern Jiangsu Province and northern Zhejiang Province. This region is one of China’s most developed, dynamic, densely populated and concentrated industrial areas. The YRDUA has grown into one of six influential world-class metropolitan areas, and it plays an important role in China’s economic and social development [22,24]. Generally, the boundary of the YRD varies according to the culture, economy, or geography considered in the different studies. The Yangtze River Delta Regional Plan issued by the State Council of China in May 2010 showed that the YRDUA covered a total of 30 cities including Shanghai, 13 cities in Jiangsu, 11 cities in Zhejiang and 5 cities in Anhui Provinces and excluded 11 cities in Anhui Province (Haozhou, Huaibei, Suzhou, Fuyang, Bengpu, Lu’an, Anqing, Chizhou, Tongli, Xuancheng and Huangshan). However, to discuss the background warming rate and differentiate the UHI contribution, this study referred to the YRDUA as comprising all regions of Shanghai, Jiangsu, Zhejiang and Anhui Provinces (Figure 1).

### 2.2. Data Set

The time series of the daily average, minimal (nighttime) and maximal (daytime) air temperature observed at 41 meteorological stations covering long-term consistent observation are used to analyze the variability of the warming rate in the YRDUA. The data are obtained from the China Meteorological Data Sharing Service System issued by the National Meteorological Information Center of China Meteorological Administration. The data have widely been used to study the climate change in China [25,26]. The stations are selected by considering the locations, lengths and completeness of the records so that the entire YRDUA can be uniformly covered. The locations of the YRDUA and selected stations are presented in Figure 1. All the time series data represent 1957 to 2013, with no missing years. The basic information, including latitude, longitude, altitude, city type and urban size, are presented in Table 1. All stations are located in the plain area with the highest elevation less than 200 m. Therefore, the effect of elevation on warming rate is negligible.

According to the classification standard of the report “Green book of small- and medium-sized cities: Annual report on development of small- and medium-sized cities in China”, we categorize the cities in the YRDUA into five levels according to population size: huge city (I: more than 10 million people), megalopolis (II: 3 million to 10 million people), large city (III: 1 million to 3 million people), medium city (IV: 5 hundred thousand to 1 million people), small city (V: less than 5 hundred thousand people).

**Table 1:** Information on 41 meteorological stations in the YRDUA.

Number	Name	Latitude	Longitude	Altitude(m)	Province	City	City Type	Urban Size
1	Longhua	31°10′	121°26′	2.6	Shanghai	Shanghai	A	I
2	Wuxi	31°35′	120°21′	5.2	Jiangsu	Wuxi	B	III
3	Liyang	31°26′	119°29′	7.7	Jiangsu	Liyang	C	IV
4	Changzhou	31°53′	119°59′	4.4	Jiangsu	Changzhou	B	III
5	Nantong	31°59′	120°53′	6.1	Jiangsu	Nantong	B	III
6	Dongtai	32°52′	120°19′	4.3	Jiangsu	Dongtai	C	IV
7	Gaoyou	32°48′	119°27′	5.4	Jiangsu	Gaoyou	C	IV
8	Nanjing	32°00′	118°48′	7.1	Jiangsu	Nanjing	A	II
9	Sheyang	33°46′	120°15′	2.0	Jiangsu	Sheyang	C	V
10	Huai’an	33°38′	119°01′	14.4	Jiangsu	Huai’an	B	III
11	Xuyi	32°59′	118°31′	40.8	Jiangsu	Xuyi	C	V
12	Ganyu	34°50′	119°07′	3.3	Jiangsu	Ganyu	C	V
13	Xuzhou	34°17′	117°09′	41.2	Jiangsu	Xuzhou	B	III
14	Yuhuan	28°05′	121°16′	95.9	Zhejiang	Yuhuan	C	V
15	Hongjia	28°37′	121°25′	4.6	Zhejiang	Taizhou	B	III
16	Longquan	28°04′	119°08′	195.5	Zhejiang	Longquan	C	V
17	Lishui	28°27′	119°55′	59.7	Zhejiang	Lishui	B	V
18	Quzhou	29°00′	118°54′	82.4	Zhejiang	Quzhou	B	V
19	Shipu	29°12′	121°57′	128.4	Zhejiang	Xiangshan	C	V
20	Yinxian	29°52′	121°34′	4.8	Zhejiang	Ningbo	B	III
21	Shengxian	29°36′	120°49′	104.3	Zhejiang	Shengzhou	C	IV
22	Jinhua	29°07′	119°39′	62.6	Zhejiang	Jinhua	B	IV
23	Dinghai	30°02′	122°06′	35.7	Zhejiang	Zhoushan	B	IV
24	Pinghu	30°37′	121°05′	5.4	Zhejiang	Pinghu	C	IV
25	Hangzhou	30°14′	120°10′	41.7	Zhejiang	Hangzhou	A	II
26	Huzhou	30°52′	120°03′	7.4	Zhejiang	Huzhou	B	III
27	Chun’an	29°37′	119°01′	171.4	Zhejiang	Chun’an	C	V
28	Tunxi	29°43′	118°17′	142.7	Anhui	Huangshan	B	V
29	Ningguo	30°37′	118°59′	89.4	Anhui	Ningguo	C	V
30	Anqing	30°32′	117°03′	19.8	Anhui	Anqing	B	IV
31	Wuhu	31°09′	118°35′	21.1	Anhui	Wuhuxian	B	IV
32	Chaohu	31°37′	117°52′	22.4	Anhui	Chaohu	C	V
33	Hefei	31°47′	117°18′	27.0	Anhui	Hefei	A	III
34	Huoshan	31°24′	116°19′	86.4	Anhui	Huoshan	C	V
35	Lu’an	31°45′	116°30′	60.5	Anhui	Lu’an	B	V
36	Chuxian	32°18′	118°18′	27.5	Anhui	Chuzhou	B	IV
37	Bengbu	32°55′	117°23′	21.9	Anhui	Bengbu	B	IV
38	Shouxian	32°33′	116°47′	22.7	Anhui	Shouxian	C	V
39	Fuyang	32°52′	115°44′	32.7	Anhui	Fuyang	B	IV
40	Haozhou	33°52′	115°46′	37.7	Anhui	Haozhou	B	V
41	Dangshan	34°26′	116°20′	44.2	Anhui	Dangshan	C	V

A: provincial capital; B: prefecture-level city; C: county-level city. I: huge city; II: megalopolis; III: large city; IV: medium-sized city; V: small city.

The urbanization rates defined as a percentage of urban population to total population, populations and areas of the built-up districts were obtained from statistical yearbooks, including the Shanghai Statistical Yearbook 2013 [27], Jiangsu Statistical Yearbook 2013 [28], Zhejiang Statistical Yearbook 2013 [29], Anhui Statistical Yearbook 2013 [30], and the Chinese City Statistical Yearbook 2013 [31].

### 2.3. Data Analysis

Statistical analyses, including averaging, linear and non-linear fitting, and regression and correlation analyses, are performed to characterize the UHI and elucidate the affecting factors of UHI using the Statistical Program for Social Sciences (SPSS) 17.0 software (IBM Corp., Chicago, IL, USA). The warming rate was obtained through the linear fitting of the yearly average, minimal and maximal air temperature *vs.* year, which was widely used for the time series analysis of meteorological parameters [32,33,34]. The significance levels are reported as significant if *p* < 0.05. Because the number of cities of the five urban sizes differed substantially, differences in the parameters among the five urban sizes are assessed with independent sample *t*-tests (*p* < 0.05).

## 3. Results and Discussion

### 3.1. Spatial Distribution of the Warming Rate

The linear fitting of the yearly average, minimal and maximal air temperature *vs.* year showed that a significant climate warming rate was found for all 41 stations in the YRDUA (Table 2). The warming rate of average, minimal and maximal air temperature ranged from 0.108 °C/decade in Chun’an to 0.483 °C/decade in Shanghai, from 0.061 °C/decade in Quzhou to 0.521 °C/decade in Shanghai, and from 0.049 °C/decade in Chun’an to 0.433 °C/decade in Shanghai, respectively. The average warming rate of average, minimal and maximal air temperature was 0.229 ± 0.074, 0.270 ± 0.096 and 0.192 ± 0.088 °C/decade of these observation stations, respectively. Overall, the order of the warming rate was minimal > average > maximal air temperature. In addition, the linear fitting of average and minimal air temperature *vs.* year was significant for all 41 stations (*p* < 0.05) (Table 2). However, the linear fitting of maximal air temperature *vs.* year was not statistically significant for some stations (Table 2). Therefore, the warming trend of nighttime was more marked than that of daytime. Similarly, the magnitude of the mean nighttime UHI from the long-term measurements was found to be much stronger than the daytime UHI during all seasons in Athens (Greece) [35]. The main reason for the day-night asymmetry of urban warming is believed to be in the vertical stability of the atmospheric boundary layer. In the daytime, the strong vertical mixing results in diffusion of excessive heat in cities and reduces temperature anomaly. In the nighttime, the thermal anomaly in cities is confined to a shallow layer, where strong inversion develops in the rural area.

**Table 2:** Warming rate (°C/decade), determination coefficient (*r*^2^) and significance level (*p*) of the linear fit of the air temperature *vs.* year from 1957 to 2013 for all 41 stations.

Station Name	Average Air temperature			Minimal Air Temperature			Maximal Air Temperature		
	Warming Rate	*r*^2^	*p*	Warming Rate	*r*^2^	*p*	Warming Rate	*r*^2^	*p*
Longhua	0.483	0.71	<0.001	0.521	0.67	<0.001	0.433	0.64	<0.001
Wuxi	0.358	0.54	<0.001	0.415	0.58	<0.001	0.325	0.47	<0.001
Liyang	0.289	0.47	<0.001	0.334	0.48	<0.001	0.235	0.32	<0.001
Changzhou	0.292	0.52	<0.001	0.341	0.59	<0.001	0.239	0.35	<0.001
Nantong	0.272	0.41	<0.001	0.263	0.33	<0.001	0.259	0.35	<0.001
Dongtai	0.207	0.31	<0.001	0.267	0.38	<0.001	0.139	0.15	<0.005
Gaoyou	0.291	0.46	<0.001	0.370	0.55	<0.001	0.187	0.23	<0.001
Nanjing	0.293	0.50	<0.001	0.332	0.50	<0.001	0.165	0.17	<0.005
Sheyang	0.256	0.41	<0.001	0.282	0.38	<0.001	0.220	0.29	<0.001
Huai’an	0.267	0.46	<0.001	0.302	0.48	<0.001	0.143	0.14	<0.005
Xuyi	0.206	0.35	<0.001	0.329	0.56	<0.001	0.063	0.03	>0.05
Ganyu	0.250	0.37	<0.001	0.392	0.49	<0.001	0.134	0.14	<0.005
Xuzhou	0.258	0.44	<0.001	0.384	0.60	<0.001	0.076	0.04	>0.05
Yuhuan	0.186	0.35	<0.001	0.162	0.27	<0.001	0.211	0.39	<0.001
Hongjia	0.281	0.47	<0.001	0.318	0.44	<0.001	0.291	0.46	<0.001
Longquan	0.137	0.23	<0.001	0.152	0.25	<0.001	0.094	0.06	>0.05
Lishui	0.162	0.27	<0.001	0.199	0.38	<0.001	0.176	0.20	<0.001
Quzhou	0.110	0.18	<0.005	0.061	0.09	<0.05	0.158	0.17	<0.001
Shipu	0.191	0.35	<0.001	0.162	0.27	<0.001	0.251	0.40	<0.001
Yinxian	0.337	0.56	<0.001	0.367	0.53	<0.001	0.377	0.51	<0.001
Shengxian	0.185	0.33	<0.001	0.150	0.24	<0.001	0.221	0.29	<0.001
Jinhua	0.214	0.34	<0.001	0.292	0.51	<0.001	0.170	0.16	<0.005
Dinghai	0.183	0.35	<0.001	0.091	0.11	<0.05	0.271	0.47	<0.001
Pinghu	0.268	0.42	<0.001	0.251	0.32	<0.001	0.242	0.43	<0.001
Hangzhou	0.335	0.60	<0.001	0.339	0.62	<0.001	0.308	0.44	<0.001
Huzhou	0.233	0.42	<0.001	0.221	0.35	<0.001	0.236	0.32	<0.001
Chun’an	0.108	0.13	<0.01	0.210	0.37	<0.01	0.049	0.02	>0.05
Tunxi	0.193	0.38	<0.001	0.228	0.48	<0.001	0.151	0.13	<0.01
Ningguo	0.177	0.32	<0.001	0.195	0.35	<0.001	0.163	0.16	<0.005
Anqing	0.257	0.44	<0.001	0.326	0.54	<0.001	0.184	0.20	<0.001
Wuhu	0.184	0.29	<0.001	0.206	0.35	<0.001	0.274	0.38	<0.001
Chaohu	0.158	0.18	<0.005	0.138	0.11	<0.05	0.308	0.43	<0.001
Hefei	0.241	0.41	<0.001	0.229	0.36	<0.001	0.201	0.23	<0.001
Huoshan	0.149	0.20	<0.005	0.202	0.33	<0.001	0.141	0.09	<0.05
Lu’an	0.222	0.34	<0.001	0.316	0.57	<0.001	0.194	0.17	<0.001
Chuzhou	0.243	0.35	<0.001	0.346	0.49	<0.001	0.179	0.18	<0.001
Bengbu	0.217	0.34	<0.001	0.353	0.60	<0.001	0.084	0.04	>0.05
Shouxian	0.177	0.32	<0.001	0.250	0.49	<0.001	0.088	0.05	>0.05
Fuyang	0.158	0.25	<0.005	0.198	0.31	<0.001	0.071	0.03	>0.05
Bozhou	0.237	0.33	<0.001	0.394	0.54	<0.001	0.078	0.03	>0.05
Dangshan	0.119	0.13	<0.01	0.163	0.16	<0.005	0.097	0.06	>0.05

**Figure 2:**
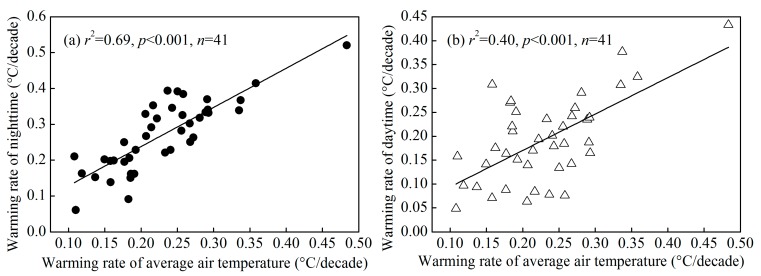
Significant linear relationship between the warming rates of average, minimal (**a**) and maximal (**b**) air temperature in in the YRDUA.

In this study, the warming rates of minimal and maximal air temperature were significantly correlated to that of average air temperature (Figure 2). In order to focus the aim of assessing the factors that affect the warming rate and differentiating the UHI contributions to the warming rate, we will pay our emphasis on the warming rate of average air temperature in the following results and discussion. A more detailed investigation for the interpretation of the difference of the warming rate of nighttime and daytime will be presented in the future study, which actually reflects the different mechanisms that determine nighttime and daytime UHI in the city.

This average warming rate of average air temperature was consistent with the results observed in other urban regions of China [34,36,37], some large US cities [38], and some Europe cities [35,39]. For example, the warming rates during 1961–2000 reached 0.32 °C/decade and 0.31 °C/decade at the Beijing station and Wuhan station, respectively [36]. Thirty-eight of 50 US cities experienced an average warming rate of 0.30 °C/decade between 1951 and 2000 [38]. However, our results were markedly lower than the warming rate from 0.502 to 0.904 °C/decade of rural and urban stations in East China for the period of 1981–2007 [19], for which the warming rate was more significant over the past 30 years due to the rapid urbanization during this period in East China [40].

**Figure 3:**
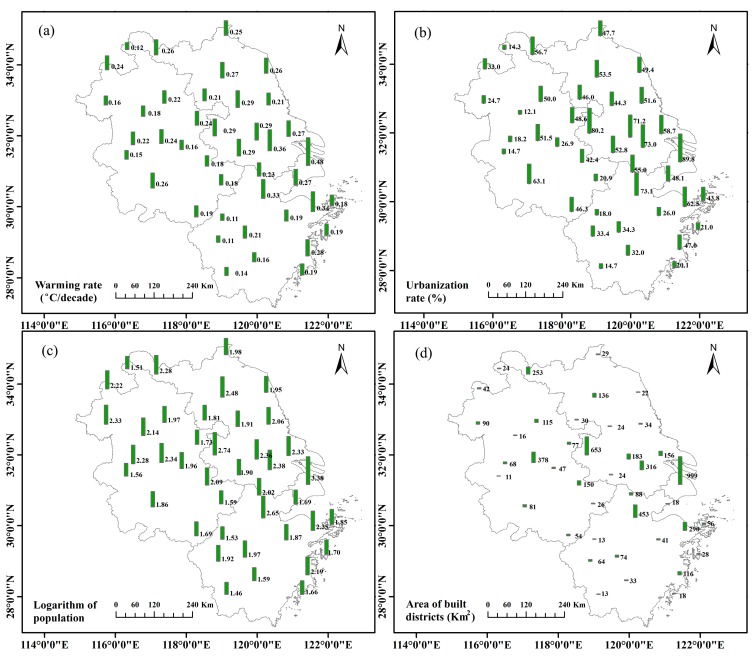
Spatial distribution of the warming rate of average air temperature (**a**), urbanization rate (**b**), logarithm of the population (**c**), and built-up areas (**d**).

In the YRDUA, the warming rate of average air temperature had significant spatial variability although this variability was not continuous because most stations were located in the urban region (Figure 3a). From Shanghai to Jiangsu and Zhejiang Provinces and further to Anhui Province, the warming rate decreased gradually as the distance from Shanghai increased. In addition, there were two small peaks around Nanjing and Hangzhou. The weak warming rates mainly occurred at small- or medium-sized city stations located in Anhui Province and in southern Zhejiang Province, where the economy is less developed and has more rural areas. Overall, the spatial pattern of the warming rate was highly consistent with those of the urbanization rate, the population, and the built-up areas (Figure 3b–d). We will further discuss the relationship between the warming rate and the urbanization rate, population, and built-up area in the section “Factors affecting the warming rate”.

### 3.2. Comparison of Warming Rates of Different Urban Sizes

**Figure 4:**
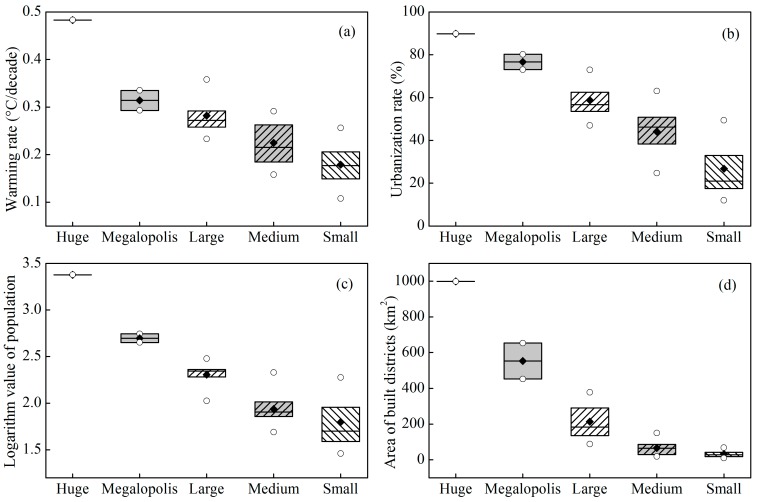
Box plot of five urban sizes for the warming rate (**a**), urbanization rate (**b**), logarithm of the population (**c**), and built-up area (**d**). The box is determined by the 25th and 75th percentiles; the values for the median (horizontal line), mean (solid diamond), minimum, and maximum (hollow circle) are also included.

**Figure 5:**
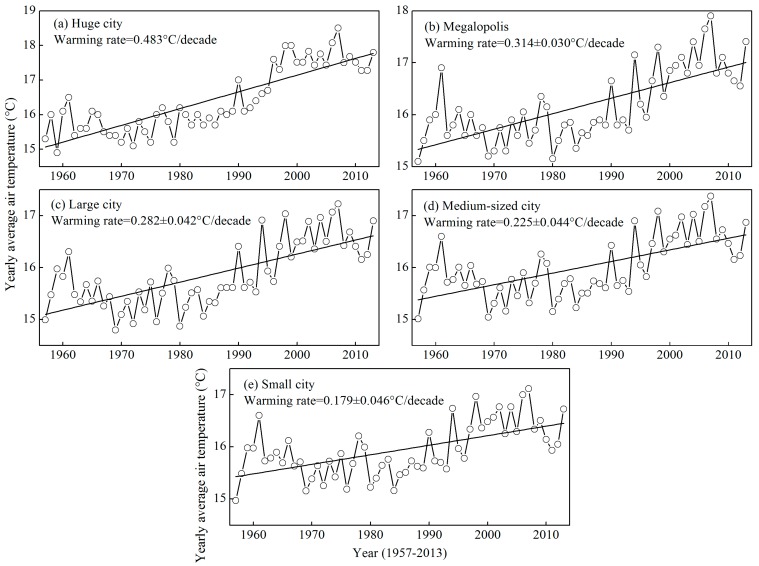
Long-term trends in the yearly average air temperature for huge city (**a**), megalopolis (**b**), large city (**c**), medium-sized city (**d**), and small city (**e**) and the linear fit (straight lines) of the yearly average air temperature *vs.* year. The warming rate is the average value of several stations with the same urban size.

The variations in the warming rate of average air temperature, urbanization, logarithm of the population, and built-up area for five urban sizes are shown as boxplots in Figure 4. Figure 5 presents the time series of the yearly average air temperature of five urban sizes for the period of 1957–2013. The average warming rate decreased from 0.483 °C/decade in the huge city, to 0.314 ± 0.030 °C/decade in the megalopolis, 0.282 ± 0.042 °C/decade in the large city, 0.225 ± 0.044 °C/decade in medium-sized city, and to 0.179 ± 0.046 °C/decade in the small city (Figure 4a). The warming rate in huge city of Shanghai showed the most significant warming trends. Conversely, sites with the lowest warming trends are mainly located in small or medium-sized cities in Anhui Province, which has a less developed economy. The average warming rate of a huge city and megalopolis is significantly higher than that of a medium-sized city and small city, indicating that the UHI has a significant effect on climate warming (*t*-test, *p* < 0.05). The gradual decrease in the warming rate showed that the UHI effect on the climate warming decreased with the decrease in the urban size (Figure 4b–d). Similar results were observed in previous studies. For example, Yang *et al*. [19] found that the warming rates were 0.904 °C/decade in a megalopolis, 0.742 °C/decade in a large city, 0.674 °C/decade in a medium-sized city, and 0.614 °C/decade in a small city in East China using the “observation minus reanalysis” method from 1981 to 2007. However, our warming rates were markedly lower than those observed by Yang *et al*. [19]. The differences could be partially attributed to the data period. The authors used data from 1981 to 2007, but we used data from 1957 to 2013. The rapid urbanization in China started in late 1978 during the reform and opening up process [40]. Therefore, the effect of the UHI was more significant over the past 30 years than over 1957–1980.

### 3.3. Factors Affecting the Warming Rate

**Figure 6:**
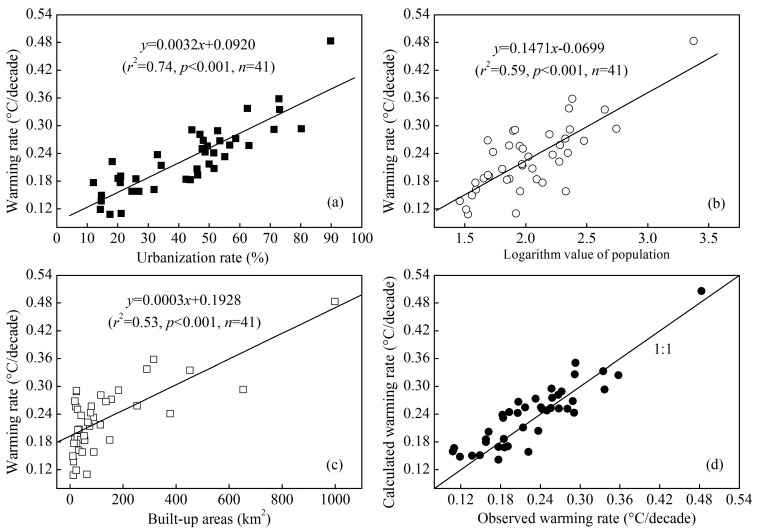
Linear relationships between the urbanization rate (**a**), population (**b**), built-up areas (**c**) and the warming rate of average air temperature for all 41 stations in the YRDUA. Comparison of the observed warming rate from the fit of the yearly mean daily air temperature *vs.* year as calculated from the multiple linear regression using the urbanization rate, population, and built-up area (**d**).

Urbanization, including land use changes from non-construction land to construction land, dense building development, which alter sensible and latent heat fluxes, has a significant impact on climate warming. The study of the temperature trends at urban and non-urban stations between 1991 and 2000 showed that the UHI due to land use changes was significant at urban stations [41]. In addition, the temperature series were markedly affected by the UHI for most cities with populations of over 100,000 in China through the analysis of the homogenized annual mean surface air temperature data over 1954−2002 [17]. The urbanization rate, land use change and population are the main indices of urbanization. Therefore, we select the urbanization rate, population and built-up area as UHI indices that affect climate warming. Figure 6 shows that significant positive correlations are found between the urbanization rate, population, built-up areas and the climate warming rate of average air temperature. Of the three indices, the urbanization rate explains the most (74%) to the climate warming rate.

Previous studies mostly emphasized the effects of land use change, the expansion of built-up land, population increases and gross domestic product (GDP) on UHI intensity (the temperature difference between urban and rural stations) for a specific region or city [42,43,44,45]. Oke [10] found a linear relationship between the nocturnal heat-island intensity and the logarithm of the city population. Significantly positive correlations were observed between the UHI intensity and the total population, GDP, and area of paved roads in Shanghai [42]. However, a global study of 419 large cities found no relation between UHI intensity and population density or city size, but it emphasized the key role of urban vegetation cover and urban activities [46]. Our study presented significant correlations between the warming rate and urbanization rate, population, and built-up area in the YRDUA from a wide spatial perspective. Similarly, a significant correlation between the warming rate and the logarithm of the population was found for Japanese cities [11].

Multiple linear regression (Equation (1)) showed that the urbanization rate, population, and area of built-up districts could explain more than 80.7% of the climate warming variability. In addition, the observed and calculated warming rates were distributed along the 1:1 line (Figure 6d), indicating that the warming rate could be equally well explained by the urbanization rate, population, and built-up area.

(1) $$\begin{array}{c} y=0.003\;x_{1}+8.048\times 10^{-5}\;x_{2}-6.008\times 10^{-5}\;x_{3}+0.105\\ \left(r^{2}=0.807,\;p<0.001,\;n=41\right) \end{array}$$

where *y* is the warming rate of average air temperature; and *x*_1_, *x*_2_, and *x*_3_ are the urbanization rate, population, and built-up areas, respectively.

The multiple linear regression revealed that the warming amount (the intercept of linear regression) that cannot be explained by the urbanization rate, population, and built-up area was 0.105 ± 0.014 °C/decade, which is lower than the former average warming rate of 0.179 ± 0.046 °C/decade for the small city. Therefore, a marked climate warming occurred due to the UHI, even for the small city, which further confirms Oke’s conclusion that a city of 1000 people could have an UHI effect [10]. Similarly, a study in the South Tyrol Province, northern Italy showed pronounced UHI effects for cities smaller than 1 km^2^ [47]. In addition, this value is very similar to the two lowest warming rates of 0.108 °C/decade in the small city of Chun’an and 0.110 °C/decade in the small city of Chuzhou (Table 2); these rates are representative of rural stations. Previous studies estimated the UHI effect by comparing observed temperatures at urban stations with those at the surrounding rural stations or by comparing the temperature between two urban-sized stations at the regional scale or between two regions at large scales [5,6,36]. Considering that this value is very close to the lowest warming rate in the smallest city similar to rural region, we therefore assume 0.105 ± 0.014 °C/decade is the background warming rate (*i.e.*, without urbanization) of this region. This warming rate is very similar to the background warming rate of 0.12 °C/decade near Hong Kong over 1971–2010 [32]. In addition, the global warming rate from 1951 to 2012 ranged from 0.08 to 0.14 °C/decade with an average value of 0.12 °C/decade [1].

Subtracting this background warming rate from the observed warming rates of the five urban sizes yields the estimated contribution of urbanization to the climate warming rate. The average warming rate attributable to urbanization was 0.124 ± 0.074 °C/decade in the YRDUA. This value is much larger than the previous estimate of 0.05 °C/decade for Southeast China from 1979 to 1998 [5] but is very similar to the 0.11 °C/decade for North China [48] and 0.10 °C/decade for China when considering the annual land-based data for a long period (1951–2004) relative to the sea-surface temperature [18]. In addition, the UHI-induced warming rate of 0.368 °C/decade for the largest city (Shanghai) was consistent with the value of 0.40 °C/decade observed in other studies [19]. Therefore, the calculated warming rate caused by UHI in our study is reliable and acceptable.

The relative contributions of the UHI to the warming rates of average air temperature in huge cities, megalopolises, large cities, medium-sized cities, and small cities were 78.3%, 66.4%, 62.1%, 51.5%, and 37.1%, respectively. Our results on the relative contribution of the UHI to climate warming are consistent with previous studies. Ren *et al*. [36] found that urbanization-induced warming for Beijing (Wuhan) was significant and accounted for 80.4% (64.5%) of the warming over 1961−2000 and 61.3% (39.5%) of the warming over 1981−2000. Similarly, the contribution of urbanization to the warming trend was higher than 40% (40%–50%) in Hong Kong [32]. The results of this study and many other studies showed that urbanization significantly enhances local climate warming. In the future few decades, the effect of UHI due to urbanization on the local climate will further aggravate with the implementation of China’s National New-type Urbanization Plan [40]. The warming rate due to the UHI and its contributions to the climate warming in the fifth report of the IPCC can still be regarded as conservative in the urban agglomeration region. Some studies have suggested that the significant contribution of urbanization to temperature changes might be comparable to that of GHG emission for metropolises and large cities [19]. Of course, the urban area only represents a very limited area of the Yangtze River Delta, the effect of UHI due to urbanization on the local climate might vary with the location of meteorological station as the study in Beijing Municipality showed [49]. In addition, the seasonal characteristics of UHI as observed in other regions [49] should be included in the future study to elucidate the fine effect due to the urbanization in the YRD.

## 4. Conclusions

China has experienced rapid economic growth and urbanization [40], and the YRDUA has become one of six influential world-class metropolitan areas in recent decades. Our analysis of daily average, minimal and maximal air temperature observations at 41 stations in the YRDUA over 1957−2010 has revealed significant long-term warming due to the background warming and the UHI. The warming rate ranging from 0.108 to 0.483 °C/decade for average air temperature is generally consistent with the warming trend of other urban regions in China and in other urban agglomerations worldwide [5,11]. Significant positive correlations were found between three urbanization factors (urbanization rate, population, and built-up area) and the warming rates. All three factors could explain more than 80% of the variability in the warming rate. Our attempt to estimate the contribution of the UHI to the observed warming based on multiple linear regression and warming rates suggests that 37.1%–78.3% of the warming in the last few decades could be explained by local urbanization at various urban sizes. The results of this study showed that urbanization significantly enhanced local climate warming. However, more detailed physical explanations of the UHI’s role in the warming rate are needed in future studies. In this study, social-economic factors of urbanization, such as the urbanization rate, population and built-up area have been emphasized. However, they are only indirectly related to the warming rate of UHI. The actual physical factors, including reduced evapotranspiration of the impervious surfaces with scarce vegetation cover, complicated changes in the surface energy balance between impervious surfaces and vegetation, and anthropogenic heat release from automobiles, power plants, air conditioners should be elucidated in the future study.
